# Expression of Recombinant Human Alpha-Lactalbumin in the Milk of Transgenic Goats Using a Hybrid Pomoter/Enhancer

**DOI:** 10.1155/2014/281031

**Published:** 2014-01-09

**Authors:** Yu-Guo Yuan, Liyou An, Baoli Yu, Shaozheng Song, Feng Zhou, Liqing Zhang, Yinyin Gu, Minghui Yu, Yong Cheng

**Affiliations:** College of Veterinary Medicine and Jiangsu Co-innovation Center for Prevention and Control of Important Animal Infectious Diseases and Zoonoses, Yangzhou University, Yangzhou, Jiangsu 225009, China

## Abstract

To improve nutrient content of goat milk, we describe the construction of a vector (pBLAC) containing a hybrid goat **β**-lactoglobulin (BLG) promoter/cytomegalovirus (CMV) enhancer. We also describe the generation of transgenic goats expressing rhLA by somatic cell nuclear transfer (SCNT). Of 334 one-cell stage embryos derived from three transgenic cell lines and 99 embryos derived from non-transgenic (NT) cells surgically transferred to the oviducts of 37 recipients, two recipients delivered two kids (2%) from the non-transfected line and five recipients delivered six kids (1.8%) from transgenic cell lines, three of which died within 2 days. Compared to the NT donor cells, transfection of donor cells does not negatively affect the development of nuclear transfer embryos into viable transgenic offspring. However, the clone efficiency in cell line number 1 was lower than that in numbers 2 and 3, and in the NT lines (0.9% versus 1.9% 2.4% and 2%; *P* < 0.05). Two transgenic cloned goats expressed rhLA in the milk at 0.1–0.9 mg/mL. The mammary gland-specific expression vector pBLAC with hybrid BLG/CMV can drive the hLA gene to express *in vitro* and *in vivo*. These data establish the basis for use of a hybrid promoter/enhancer strategy to produce rhLA transgenic goats.

## 1. Introduction

The mammary gland is currently the only readily available animal bioreactor, but optimized expression vector construction is required for transgenic expression of recombinant proteins, and expression vectors still have strides to make [1, 2]. One strategy for optimized expression vectors includes using elements like insulators and matrix-attached regions, but the expression level of proteins is often modest. Construction of the targeting vector is one of the most important techniques for mammary gland bioreactors, but the currently available techniques are laborious, time consuming, and inefficient [3]. Constructs with milk protein gene loci could be an ideal strategy for high level expression of foreign genes [2, 4, 5], but it is often difficult to manipulate the large size of the vector into donor cells for nuclear transfer [3, 6]. In our previous studies [7], a hybrid milk protein promoter (goat *β*-casein, bovine *α*s1-casein, and goat *β*-lactoglobulin (BLG)) with cytomegalovirus (CMV) enhancer was constructed and the expression level of recombinant human lactoferrin in the milk of transgenic mice was up to 8.2 mg/mL, which was more effective than using a single milk protein promoter (7–40 ng/mL). Therefore, construction of a hybrid milk protein promoter with enhancer is an attractive way to improve the expression level of recombinant proteins. Although the use of a hybrid promoter/enhancer is a valuable strategy in a transgenic mouse model, it is still unknown whether it is also effective for other animals, such as goats.

Genetic modification has resulted in animals that have tremendous utility for both agriculture and medicine [8]. Practical applications of transgenic livestock include improved milk production and nutrient composition, increased growth rate, improved feed usage, improved carcass composition, increased disease resistance, enhanced reproductive performance, and increased reproductive capacity [9, 10]. Goat milk besides other milks is a significant food and nutrient source for people in many countries, with up to 55% of all milks produced in one country. To improve the nutritional value of goat milk and supply human *α*-LA for pharmaceutical research, expression of human *α*-LA in goat milk by transgenic technology appears to be promising. Alpha-lactalbumin (*α*-LA) is a 14 kDa Ca^2+^-binding milk protein synthesized in the secretory cells of lactating mammary glands [11]. Its main function is to interact with b1,4-galactosyltrans-ferase-1 (b4Gal-T1) to form lactose synthase complex, which is responsible for the production of lactose. Expression of *α*-LA in the milk increased milk production via increased lactose synthesis, which led to increased neonatal growth [12, 13]. *α*-LA accounts for 28% of the total protein in human milk but only 3% of the total protein in goat milk [14]. Furthermore, *α*-LA contains a relatively high proportion of essential amino acids and thus has physiological and nutritional significance for humans. *α*-LA may also be effective in preventing gastrointestinal infections among neonates [15]. Therefore, adding value (hLA) to goat whey proteins by application of SCNT may confer functional benefits on the health and growth of kids, creating a potential economic benefit. Furthermore, hLA is a nutritional protein in human milk, so it is beneficial to adjust the components of goat milk to resemble that of human milk to better satisfy the nutritional requirements of infants and adults. Recently, the production of transgenic cows that produced human *α*-LA in their milk was reported to improve the nutritional value of the milk [16], but the same application in transgenic goats has not been reported. In the present study, we describe the use of the hLA gene as a model for transgenic expression. A vector with goat BLG promoter combined with CMV enhancer was constructed and transfected into goat fetal fibroblast cell lines (GFFCs) for nuclear donors and the expression level of rhLA in the milk of the transgenic cloned goats was tested. These data will provide the basis for the use of a hybrid promoter/enhancer strategy for producing transgenic goats.

## 2. Materials and Methods

### 2.1. Reagents

Unless otherwise stated, all media and components were purchased from Sigma-Aldrich Corp. (St. Louis, MO, USA).

### 2.2. Transgenic Expression Vector for hLA

**Figure 1:**
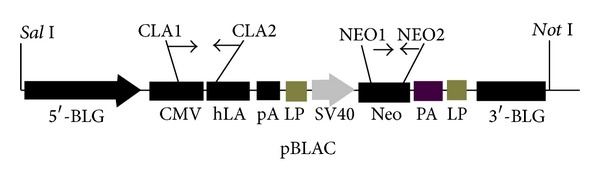
Schematic representation of pBLAC construct. 5′-BLG: goat *β*-lactoglobulin promoter; CMV: human cytomegalovirus immediate early promoter; hLA: human lactalbumin gDNA; pA: SV40 early mRNA polyadenylation; LP: LoxP sequence; SV40: simian vacuolating virus 40 promoter; Neo: neomycin resistance gene; 3′-BLG: *β*-lactoglobulin.

The genomic DNA (gDNA) sequence encoding hLA (GenBank Accession no. X05153.1), which contained exon 1-4, intron 1-3, and poly A sequence (2.36 kb), was prepared by polymerase chain reaction (PCR) using human blood DNA as the template with primer sets (sense: GCTCGAGATGAGGTT CTTTGTC CCTC; antisense: GCTCGAGTGACTTCAAAGTGGGACC). To facilitate the subcloning into pBCc [7], the *Xho* I (TAKARA) site was added to the 5′ and 3′-terminal end. The PCR reactions consisted of 94°C for 3 min in the first instance, 94°C for 40 sec, and 68°C for 4 min for 30 cycles, with a final extension of 72°C for 5 min. The resulting hLA sequence digested by* Xho *I was subcloned into the *Xho* I site of pBCc named pBLAC (Figure 1). The fragment for transfection was excised from pBLAC by *Sal* I-*Not* I (TAKARA) digestion, run on 0.8% Tris-acetate-ethylenediaminetetraacetic acid gel and purified using DNA Purification Kit (QIAGEN Co, Ltd., UK) according to the manufacturer's instructions.

### 2.3. Isolation and Transfection of Caprine Mammary Gland Epithelial Cells (CMGECs)

CMGECs were isolated from a hormone-induced lactating goat as previously described [17, 18]. For electroporation, 5 × 10^6^ CMGECs were collected at 80% confluence, mixed with 20 *μ*g DNA, transferred into a 0.2 cm cuvette (Eppendorf, San Diego, CA, USA), and subjected to one pulse of 0.2 kv and 100 *μ*s delivered by a gene pulser (Eppendorf). Cells were plated out in culture medium containing Dulbecco's modified Eagle's medium (DMEM)/F-12 (GIBCO, Carlsbad, CA, USA), 10% fetal calf serum (FCS; GIBCO), 10 *μ*g/mL insulin, 5 *μ*g/mL transferrin, 5 *μ*g/mL hydrocortisone, 200 U/mL penicillin-G, and 200 *μ*g/mL streptomycin sulfate for 48 h and selected with G418 (400 *μ*g/mL). After 20 days of selection, a number of single colonies were isolated and expanded.

### 2.4. Establishment and Transfection of GFFCs

The expression vector for hLA was transfected into GFFCs by electroporation as previously described by An et al. [19]. Briefly, the day before transfection, confluent fetal fibroblasts (at passage 1 to 2) were trypsinized, counted, and plated into six-well culture dishes to reach 80% confluency on the day of transfection. For electroporation, 1 × 10^6^ GFFCs were collected at 80% confluence, mixed with 6 *μ*g DNA, transferred into a 2 mm cuvette (Eppendorf), and subjected to one pulse of 0.4 kV, 100 *μ*s delivered by Multiporator (Eppendorf). Cells were plated out in culture medium containing DMEM/F-12, 10% fetal bovine serum (FBS), 200 U/mL penicillin-G, and 200 *μ*g/mL streptomycin sulfate for 48 h and selected with G418 (800 *μ*g/mL). After 14 days of selection, a number of transgenic single colonies were isolated, expanded, or frozen in liquid nitrogen.

### 2.5. Nuclear Transfer and Embryo Transfer

Oocytes were recovered surgically at 26–28 h after injection of LHRH by flushing the oviduct with Ham F-12 medium (GIBCO) plus 0.5% FBS. Nuclear transfer was carried out as described previously [18]. Briefly, mature oocytes with a first polar body were cultured in M16 containing 5 *μ*g/mL cytochalasin B for 30 min. After staining with 2 *μ*g/mL bisbenzimide (Hoechst 33342) for 2 min, oocytes were held with a holding micropipette and the zona pellucida was partially dissected with a fine glass needle to make a slit near the first polar body. The first polar body and adjacent cytoplasm, presumably containing the metaphase-II chromosomes, were extruded by squeezing with the same needle. Oocytes were observed under an inverted microscope equipped with epifluorescence. Oocytes still containing DNA material were excluded. Trypsinized single cells (transgenic or nontransgenic (NT)) with a smooth surface were selected and transferred into the perivitelline space of enucleated oocytes. These couplets were placed in a 0.3 M mannitol solution containing 0.1 mM MgSO_4_, 0.05 mM CaCl_2_, 0.5 mM HEPES, and 3 mg/mL bovine serum albumin (BSA) for 5 min and transferred to a chamber consisting of two electrodes overlaid with fusion solution, where they were fused by two direct current pulses (1.7 kV/cm for 40 *μ*s) delivered by an electrocell fusion manipulator (KeFa; Institute of Developmental Biology, Beijing, China). After incubation for 5 h in M16 medium, the fused couplets were incubated in M16 containing 5 *μ*M ionomycin for 5 min, cultured for 5 h in M16 containing 7.5 *μ*g/mL cytochalasin B and 2 *μ*M N-6 dimethylaminopurine prepared in M16 medium, and surgically transferred into synchronized recipients on day 0 of their estrus cycle. Recipients underwent transvaginal ultrasonographic evaluation on day 30 to record fetal development.

### 2.6. Detection of Genomic Integration of pBLAC Transgene in Transgenic Donor Cells and Cloned Goats

To detect pBLAC in transgenic donor cells and cloned goats, genomic DNA was extracted and PCR amplification was performed. The PCR product of 875 bp was amplified with CLA1 (5′-TGTTCCCATAGT AACGCCAAT-3′) and CLA2 (5′-AGCGAAACTCCACTTCAAA-3′) primers. The PCR product of 435 bp was amplified with NEO1 (5′-CACTGAAGCGGGAAGGGACTG-3′) and NEO2 (5′- GCAATATCACGGGTAGCCACG-3′) primers. The PCR reaction was 95°C for 5 min in the first instance, 94°C for 45 sec, 61°C for 45 sec, and 72°C for 30 sec for 30 cycles, with a final extension of 72°C for 10 min. The PCR products were then run in a 1% agarose gel, stained with ethidium bromide, and visualized under ultraviolet light.

### 2.7. Quantitative Real-Time PCR Assays of Copy Number

The quantitative real-time PCR was performed using the line-gene K FQD-48A PCR system with SYBR Premix Kit (Promega, WI, USA). Reactions were done in a total volume of 20 *μ*L comprising 1 *μ*L sense and antisense primer (2 *μ*M), 10 *μ*L SYBR Premix, 7 *μ*L dH_2_O, and 1 *μ*L sample of genomic DNA. All primers used for quantitative PCR are listed in Table 2; PCR was performed at 95°C for 2 min, followed by 40 cycles of 95°C for 5 s and 60°C for 30 s; a melting curve was done at the end of the amplification for the specificity. Quantification was performed with the standard curve method using five standard dilutions, in triplicate, of two goats (BC186 and BC228) gDNA ranging from 16.50 to 0.33 ng (5000 to 100 haploid genome copies) per reaction. The copy number of hLA gene in each goat was estimated; ACTIN sequence was used as endogenous gene.

### 2.8. Western Blot and Enzyme-Linked Immunosorbent Assay (ELISA) Analysis of rhLA

For detection of rhLA secreted by CMGECs, 2 × 10^5^ cells were plated into a six-well cell culture. Confluent cells were cultured to induce expression of rhLA in DMEM/F12 containing 10% FCS, 10 *μ*g/mL insulin, 5 *μ*g/mL transferrin, 5 *μ*g/mL hydrocortisone, 5 *μ*g/mL prolactin, 200 U/mL penicillin-G, and 200 *μ*g/mL streptomycin sulfate for 48 h. After induction, the medium (200 *μ*L) was collected and stored at −70°C for later use. Milk was collected from lactating transgenic female goats (BC186 and BC228; 4 months of age) 18 days after hormone induction [17]. The goats were injected subcutaneously with 10 units of oxytocin. Milk was collected by gentle massage of the mammary gland, collected in a capillary tube, and stored at −70°C until analysis. Western blots and ELISA were performed as described [12]. Milk samples from CMGECs and transgenic goats were boiled in protein denaturing solution (1 : 1) for 10 min and run on a 12% sodium dodecyl sulfate polyacrylamide gel electrophoresis (SDS-PAGE) gel. The proteins (40 *μ*g) were electrophoretically transferred to polyvinylidene fluoride membranes. After transfer, the membranes were incubated overnight in phosphate-buffered saline with 0.1% Tween-20 (TPBS) supplied with 4% BSA, followed by a 2 h incubation with 1 : 1000 (v/v) dilution of rabbit anti-hLA (Santa Cruz Biotechnology, Inc., CA, USA) antibody TPBS. The membranes were washed with TPBS and incubated with secondary goat anti-rabbit IgG-HRP (1 : 1000 dilution; Santa Cruz Biotechnology, Inc., CA, USA) for 2 h. After washing, the membranes were visualized with diaminobenzidine.

Expression of hLA in two transgenic goats grains was quantified by ELISA. A 96-well microtiter plate was coated with 50 *μ*L of rabbit anti-human LA, at a 1 : 1000 dilution with 50 mM sodium bicarbonate buffer, pH 9.6 at 4°C overnight. The plate was then equilibrated at room temperature (25°C) for 1 h, the coating solution removed, and the wells washed with PBS. Samples (50 *μ*L) at appropriate dilution and standards were prepared in TPBS, added to the wells, and incubated at 37°C for 2 h. The samples were then aspirated out and the wells washed with PBS. Secondary goat anti-rabbit IgG-HRP at a 1 : 1000 ratio was added and incubated at 37°C for 1.5 h; then the wells were washed three times with PBS-T. Fifty microliters of substrate (0.5 mg/mL diaminobenzidine, 1 mg/mL imidazole, and 1 *μ*L/mL 30% H_2_O_2_ added just before use) was then added in a dark room at 37°C. The reaction was stopped by adding 50 mL of 1 N H_2_SO_4_. The developed color was read at 490 nm on an ELISA reader (RT-6000, Rayto Life and Analytical Sciences Co., Ltd. China). The findings were calculated by comparison to a standard curve.

## 3. Results 

### 3.1. Transgenic Expression Construct and *In Vitro* Expression in CMGECs

The expression plasmid containing the hLA gDNA was fused to the CMV sequence and flanked by the untranslated regions and flanking sequences of goat BLG gene, which contained 4.2 kb of 5′ and 1.2 kb of 3′ caprine BLG flanking no-coding sequence fused to 2.36 kb of hLA gDNA with a SV40 pA (Figure 1). The plasmid construction is outlined in Section 2.

**Figure 2:**
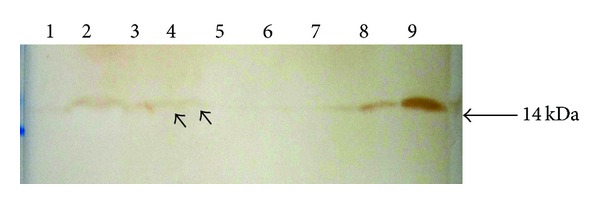
Expression of recombinant human alpha-lactalbumin (rhLA) in the supernatant of transgenic CMGECs. The result of Western blot. Lane 1: NT CMGECs control; lanes 2–5: supernatant of transgenic CMGECs; lanes 6–9: hLA.

The CMGECs were transfected with expression plasmid and selected with G418. The resistant transgenic colonies were expanded and induced to expression of protein verified by Western blot. The results showed that high-expression level (0.9–4 *μ*g/mL) of rhLA was found in eight of 45 colonies in the culture medium. A single protein band of approximately 14 kDa was shown on Western blot analysis, with a very similar migration pattern compared with the commercial human milk-derived LA (Figure 2).

### 3.2. Production of hLA Transgenic Goats

To evaluate whether the transgene could affect cloning efficiency, GFFCs were transfected with the pBLAC vector and 42 G418 resistance clones were obtained, 24 of which were confirmed to harbor the transgene by PCR. Three lines (number 1 (N17IIH27), number 2 (N17IIH15), and number 3 (N17IIH3)) were used for nuclear donor cells. Development of the embryos reconstructed with hLA transgenic and NT donor cells is summarized in Table 1. A total of 334 one-cell stage embryos reconstructed with hLA transgenic donor cells were surgically transferred to the oviducts of 25 surrogate mothers. Five pregnant goats were carried to term and naturally delivered six kids (1.8%), three of which died within 2 days of birth. In the NT group, 132 cell couplets were produced using normal fetal fibroblast cells and the fusion rate of cell couplets was 80.3%. Ninety-nine pronuclear-stage embryos were transferred into the oviducts of 12 recipient goats. Five of these (41.7%) were confirmed pregnant on day 30, and two delivered a total of two cloned kids (2%). There was no significant difference in the rate of fusion (72.3% versus 80.3%), pregnancy (48% versus 41.7%), or nuclear transfer efficiency (1.8% versus 2%) between nontransfected and transfected cell lines (*P* > 0.05). Among the three transgenic cell lines used, the cloning efficiency of number 1 was lower than that of numbers 2 and 3 (0.9% versus 1.9% and 2.4% (*P* < 0.05)), but the rate of fusion and pregnancy at 30 days was not significantly different.

Analysis of genomic DNA from six goats by PCR amplification revealed the integration of the transgene (Figure 5). The PCR products were also sent to sequencing to further confirm the transgenic events (data not shown).

**Figure 3:**
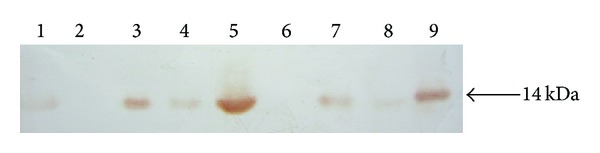
Expression of recombinant human alpha-lactalbumin (rhLA) in milk of transgenic goat. The result of western blot. Lane 1: NT goat control; lanes 2,6: blank; lanes 3, 7: milk of line# BC186 transgenic goat; lanes 4, 8: milk of line# BC228 transgenic goat; lanes 5, 9: hLA.

Three transgenic goats were induced to lactate with hormones at 4 months of age, one of which died of pneumonia on day 15 of lactation. The milk (5 *μ*L) from goats BC186 and BC228 with four and two copies of transgene was collected and analyzed using Western blot and ELISA to determine the presence of rhLA, which has a very similar migration compared with human milk-derived LA (Figure 3).

**Figure 4:**
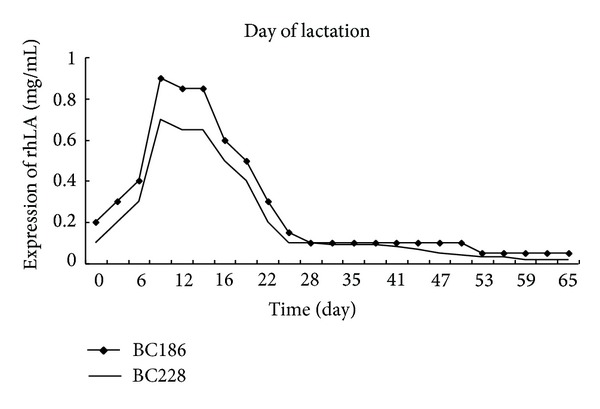
Production of human alpha-lactalbumin in transgenic goat milk during a 65 d lactation.

**Figure 5:**
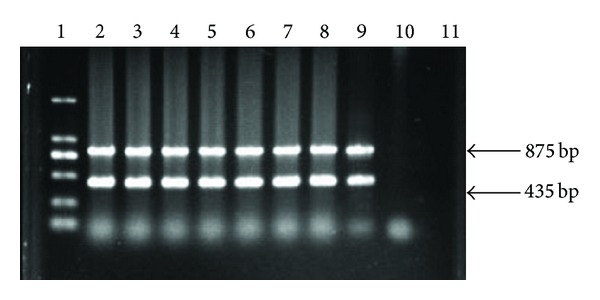
PCR analysis of transgenic goats. Lane 1: 2.0 kb ladder; lanes 2–7: transgenic goats; lanes 8-9: transgenic donor cells; lanes 10: negative control (NT goat); lanes 11: blank.

Goats were lactated with hormone and milked from day 7 through day 65 of lactation, and the milk was assayed for rhLA. Recombinant protein in the milk was present at concentrations between 0.1 and 0.9 mg/mL. Expression level of rhLA generally tended to decrease through 9 d lactation (Figure 4). This result was not in parallel with the typical goat milk production curve; goat milk yield increased throughout this period.

## 4. Discussion

The promoter of the milk protein gene (ovine or caprine beta-lactoglobulin) has been used to target high level expression of foreign protein in the milk of transgenic mice [20–23]. In our previous study, the strategy of a BLG/CMV based vector efficiently drove the human lactoferrin cDNA gene to express in the milk of transgenic mice [7]. Because the first step for the production of transgenic goats was to determine whether the expression vector can drive hLA to express in the mammary gland, CMGECs were needed to study the protein expression [18, 24]; Western blot results demonstrated that the transgenic CMGECs (eight colonies) expressed recombinant hLA at 0.9–4 *μ*g/mL in the supernatants. Recombinant hLA protein was of the expected size (14 kDa) and was identical to human milk-derived LA (Figure 2). Overall milk protein composition was similar in transgenic and NT CMGECs as estimated from SDS-polyacrylamide gels stained with Coomassie brilliant blue (data not shown). These results demonstrated that hLA gene was efficiently expressed *in vitro* in the CMGECs directed by the BLG/CMV sequence and similar results may occur *in vivo* in the transgenic goat CMGECs.

To demonstrate the hypothesis, we transfected pBLAC into fetal cells and produced transgenic goats by SCNT. Six live cloned hLA transgenic goats were produced from the transfer of 334 reconstructed embryos (1.8% efficiency) in 25 surrogates mothers; 12 resulted in pregnancies (Table 1). Three kids died within 2 days after birth due to pneumonia ordinarily reported in transgenic cloned goats (unpublished data). The remaining goats from cell lines 1 and 3 had four and two copy numbers of integrated transgene, respectively and grew without problems and were of normal size and weight. At 4 months of age, three goats were induced for milk production and one died during the first 15 days of life. The milk from the two goats (BC186 and BC228) was analyzed using a Western blot (Figure 3) and ELISA to determine the presence of rhLA (Figure 4). Recombinant protein in the milk of surviving transgenic goats from day 7 to day 65 of lactation was present at concentrations up to 0.1–0.9 mg/mL and was of the expected size (14 kDa). These data confirmed transcription and translation of the hLA gene in goats correctly and efficiently by hybrid BLG promoter/CMV enhancer. In transgenic mice, the expression level of rhLF derived by the hybrid BLG promoter/CMV enhancer was 3.4 mg/mL [7], which is more efficient than that of rhLA in goats in the present study. This difference may be caused by the different proteins or inherent factors of the different species (mice and goats). hLF and hLA were expressed by different milk protein regulatory sequences, and the high-expression level of two different foreign proteins driven by the same mouse whey acidic protein gene locus was obtained [2, 5]. However, Yu et al. [3] reported that the intron added in the coding sequence of hLF cDNA played an important role in efficient expression of rhLF and that milk levels of rhLF increased from 0.8 to 36 mg/mL, when derived by the same goat *β*-casein gene. Therefore, one reason that the hybrid BLG/CMV may not efficiently direct hLA to express in the milk of goats may be the difference of the protein gene. We also hypothesized that there was a lack of cis-acting elements responsible for the high expression of the hLA protein in the construct of pBLAC or in the hLA cDNA sequence. Liu et al. [25] and Yang et al. [6] reported that transgene expression by the construct carrying the entire hLF genomic sequence had different efficiencies in transgenic mice compared with cattle (8 mg/mL versus 3.4 mg/mL). Therefore, species may be a consideration for transgene expression in the mammary gland bioreactor. In addition, the copy number and position-dependent effect, which often occurred in the transgenic animal, should not be excluded. Although the present study demonstrated the ability of hybrid BLG/CMV to direct hLA gene to express in the milk of goats, we did not obtain goats with a high level of expression of rhLA. More studies should be pursued in this area.

The milk-specific protein *α*-LA is a key part of the lactose synthase complex in mammary epithelial cells. Expression of bovine *α*-LA in the milk of transgenic gilts and mice should result in increased milk production in early lactation and thus increased growth of the offspring [13, 26]. Transgenic cows expressing the human *α*-LA had normal milk composition, including fat and protein content [16]. In the present study, milk composition of the two transgenic goats is not changed (data not show) and expression of rhLA did not affect the health of the transgenic goats.

Transgenic animals are useful for the mass production of human therapeutic proteins. Producing livestock by SCNT combination with a transgenic approach often requires genetic modification of donor cells. The cell line of the same origin was significantly different in the development rate of the NT embryo [27, 28]. In the present study, there was a significant difference in the cloning efficiency in different transgenic cell lines when used for nuclear transfer (*P* < 0.05). However, whether the efficiency of embryos would be affected by transgenes is not yet understood. In a previous study, the development rate of bovine nuclear transfer embryos for nontransfected and transfected donor cells was similar [29] because transfection of donor cells did not affect the development of nuclear transfer embryos [30]. In contrast, the blastocyst rate of reconstructed embryos from transgenic cells was significantly lower than that from normal cells [31, 32]. In the present study, we compared developmental rates of goat embryos reconstructed with transgenic and NT donor cells. The results of these experiments indicate that transgenes have no effect on the ability of embryos to develop to term (*P* > 0.05). The pregnancy rate of surrogate mothers on day 35 for the transgenic group was slightly higher than for the NT group (48% versus 41.7%), but the fusion rate (72.3% versus 80.3%) and clone efficiency (1.8% versus 2%) were slightly lower. The clone efficiency was similar to the results reported using transgenic or NT donor cells and oocytes matured *in vivo* [3, 19, 33, 34]. In our previous studies [18], when the CMGECs were used for nuclear donor cells, the clone efficiency was 2%, similar to the result obtained with fetal fibroblast cells. These results may indicate that CMGECs have the same cloning efficiency as fetal fibroblast cells and may be another cell source for transgenic modification.

In conclusion, transgenic goats that contained the hLA gene were produced by SCNT and a vector with a hybrid promoter and enhancer was able to drive the hLA gene to express  *in vitro* and *in vivo*. In addition, transfection of donor cells had no effect on the ability of nuclear transfer embryo development.

## Figures and Tables

**Table 1:** Production of transgenic cloned goats.

Transgene	Donor cell source	No. (%) fused	No. embryos transferred	No. recipients	No. (%) pregnant (30 d)	No. % offspring
hLA	1 (N17 ΙΙ H27)	105	104	7	3 (42.9)	1 (0.9)^a^
	2 (N17 ΙΙ H15)	107	105	8	4 (50)	2 (1.9)^ab^
	3 (N17 ΙΙ H3)	129	125	10	5 (50)	3 (2.4)^ab^

Total		341 (72.3)	334	25	12 (48)	6 (1.8)^c^
NO		106 (80.3%)	99	12	5 (41.7)	2 (2)^c^

Means with different letters (a–c) are significantly different (*P* < 0.05).

**Table 2:** The primers for actin and hLA.

Actin F	CTTCCTTCCTGGGTGAGTGAGA
Actin R	ACAGCACCGTGTTGGCGTAAA

hLA F	GCATTATGCCCAGTACATGACCTTA
hLA R	CCGTGAGTCAAACCGCTATCCA
